# Optimization of banana pseudostem ash catalyst for biodiesel synthesis from crude palm oil

**DOI:** 10.1016/j.mex.2026.104037

**Published:** 2026-07-08

**Authors:** Meriatna, Husni Husin, Yuliana Sy, Medyan Riza, M. Faisal, Leni Maulinda, Pocut Nurul Alam, Muhammad Lathiful Yazil, Fikri Hasfita

**Affiliations:** a Doctoral Program of Engineering Studies, Universitas Syiah Kuala, Banda Aceh, 23111, Indonesia; b Department of Chemical Engineering, Faculty of Engineering, Universitas Syiah Kuala, Banda Aceh, 23111, Indonesia; c Department of Chemical Engineering, Faculty of Engineering, Universitas Malikussaleh, Lhokseumawe, 24352, Indonesia

**Keywords:** Catalyst, Biodiesel, Crude palm oil, Banana pseudostem ash, Musa paradisiaca Linn. Var. Awak, Response surface methodology

## Abstract

This study reports the optimization of a heterogeneous green catalyst derived from banana pseudostem ash (BPSA) of Musa paradisiaca var. Awak for biodiesel synthesis from crude palm oil (CPO). The catalyst was prepared by calcination at 600, 700, and 800 °C, characterized using standard analytical techniques, and applied in transesterification. Process optimization examined methanol to oil molar ratio (1:4-1:12), catalyst loading, and reaction time. Excess molar ratio (>1:6) reduced yield due to phase separation and limited catalyst–reactant contact. Optimal conditions 2 wt% catalyst, 1:6 oil to methanol ratio, and 120 min gave a maximum FAME yield of 99.84 wt%. GC MS identified major components as methyl oleate (59.48%), methyl palmitate (29.65%), methyl stearate (6.90%), methyl linoleate (2.27%), and methyl myristate (1.70%). The biodiesel showed density 866 kg m⁻³, kinematic viscosity 3.26 cSt, and cetane number 63.6, meeting SNI 7182:2015 and ASTM D6751 standards. BPSA from Musa paradisiaca var. Awak proves a promising, sustainable, and cost effective heterogeneous catalyst for green biodiesel production from CPO.

• Musa paradisiaca var. Awak

• Method for preparation and characterizing of Green Catalysts

•Optimization and sustainability

## Graphical abstract

**Figure:**
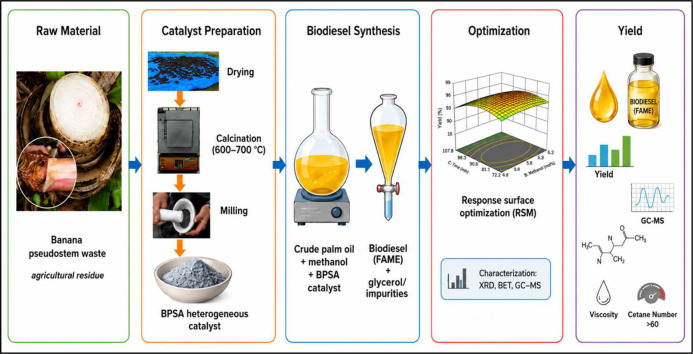

## Specifications table

**Table:** 

**Subject area**	Energy
**More specific subject area**	*Biofuel, heterogeneous catalyst, green energy*
**Name of your method**	*API gravity blend ratio,* combusted, calcined, *catalyst characterization, transesterification, Physicochemical properties*
**Name and reference of original method**	*Top of Form* *Bottom of Form* *Coconut husk ash as heterogeneous catalyst for biodiesel production from crude palm oil*
**Resource availability**	*Biofuel, Heterogeneous catalyst*

## Background

Biodiesel has been increasingly recognized as a sustainable alternative to fossil diesel because of its biodegradability, low toxicity, and capability to reduce net greenhouse gas emissions [1,2]. In Indonesia, national programs such as the B30 biodiesel mandate have been implemented to strengthen energy security, decrease dependence on imported fossil fuels, and support climate mitigation efforts. Biodiesel is typically synthesized via transesterification of triglycerides in vegetable oils such as crude palm oil (CPO) using short-chain alcohols (commonly methanol) and a suitable catalyst[3] producing fatty acid methyl esters (FAME) and glycerol as main products [4].

Catalyst selection significantly influences reaction efficiency, yield, and overall process economics. Homogeneous base catalysts such as KOH, NaOH, and NaOCH₃ offer high activity and low cost but pose challenges in product separation, equipment corrosion, and wastewater generation. Consequently, heterogeneous catalysts-including CaO[5], K_2_O[6], MgO[7], zeolites[8], and biomass-derived materials-have attracted growing interest for their simplicity in recovery, reusability, and environmental compatibility [9].

Potassium-rich agricultural wastes have recently been reported as effective precursor materials for heterogeneous catalysts in transesterification reactions. During thermal conversion, potassium-containing compounds in biomass transform into catalytically active potassium oxide species that enhance biodiesel formation. This insight has spurred the development of renewable, low-cost catalysts derived from agricultural residues such as potato peels, sugarcane bagasse, and citrus waste, showing promising performance.

Among various biomass sources, the banana pseudostem (BPS) a lignocellulosic by-product abundantly available from banana cultivation in Southeast Asia is an underutilized resource with high mineral content. Upon controlled combustion, BPS generates ash rich in potassium compounds, which can be converted into active K₂O species through calcination. While biomass-derived ash catalysts have been explored previously, systematic research on the influence of activation parameters, such as calcination temperature, on catalytic performance remains limited [10].

This study establishes a methodological framework for preparing and optimizing a heterogeneous catalyst from banana pseudostem ash (BPSA) of Musa paradisiaca Linn. var. Awak for biodiesel production from CPO. The approach combines controlled combustion and calcination of the biomass with comprehensive characterization via XRD and BET analyses to evaluate structural and chemical properties. In addition, response surface methodology (RSM) was applied to optimize key transesterification parameters and analyze interactions among process variables efficiently [11].

The main objective is to identify optimal catalyst preparation conditions, particularly combustion and calcination temperatures, to generate active, stable catalysts derived from banana pseudostem waste. Ash catalysts calcined at 600 and 700 °C were tested for biodiesel synthesis from CPO. By integrating catalyst synthesis, characterization, and statistical optimization, this research offers a reproducible and sustainable approach to converting agricultural waste into efficient heterogeneous catalysts, thereby advancing circular economy principles and green biodiesel production.

Although several biomass-derived ash catalysts have been reported for biodiesel production, most previous studies have focused on common agricultural residues and have not systematically addressed banana pseudostem ash from *Musa paradisiaca* Linn. var. Awak as a potassium-rich heterogeneous catalyst for crude palm oil transesterification. The novelty of this study lies in the integrated methodological framework that combines the controlled preparation of banana pseudostem ash, the evaluation of calcination temperature, XRD- and BET-based catalyst characterization, response surface optimization of transesterification variables, biodiesel quality assessment, and the evaluation of catalyst reusability. This approach provides a reproducible route for converting an underutilized regional agricultural waste into an efficient green catalyst for biodiesel production.

## Method details

### Materials

A waste banana pseudo-stem (BPS) from the Awak cultivar (1–1.5 years old) was obtained from local farmers in North Aceh District, Aceh Province, Indonesia. The chemicals used included hydrochloric acid (HCl, 37%, Merck), palm oil from Banda Aceh, potassium hydroxide (KOH, 99%, Merck), sodium hydroxide (NaOH, 99%, Merck), phosphoric acid (H₃PO₄), phenolphthalein, filter paper, and distilled water.

### BPSA catalyst preparation

**Fig. 1:**
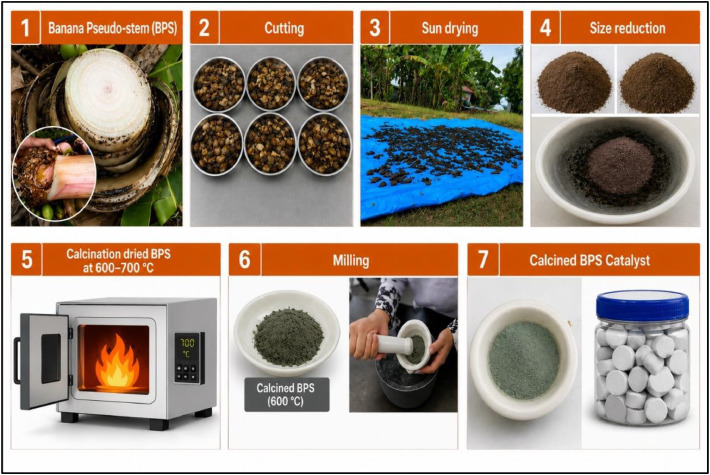
Flow diagram of BPSA catalyst preparation.

BPS derived from Musa paradisiaca L. var. Awak was washed with running water and sun-dried for about three days to reduce the moisture content to below 10%. The dried material was then cut into small pieces (approximately 2 cm) and further dried in a Memmert oven at 105 °C until a constant mass was obtained. The dried BPS was subsequently combusted to ash, ground to pass a 100-mesh sieve, and calcined in a tubular furnace (Type 21100 Firsty) at 600-800 °C for 4 h. The calcined samples were stored in a desiccator until use. The catalyst preparation procedure is illustrated in Fig. 1.

### BPS catalyst characterization

#### X-ray diffraction analysis

**Fig. 2:**
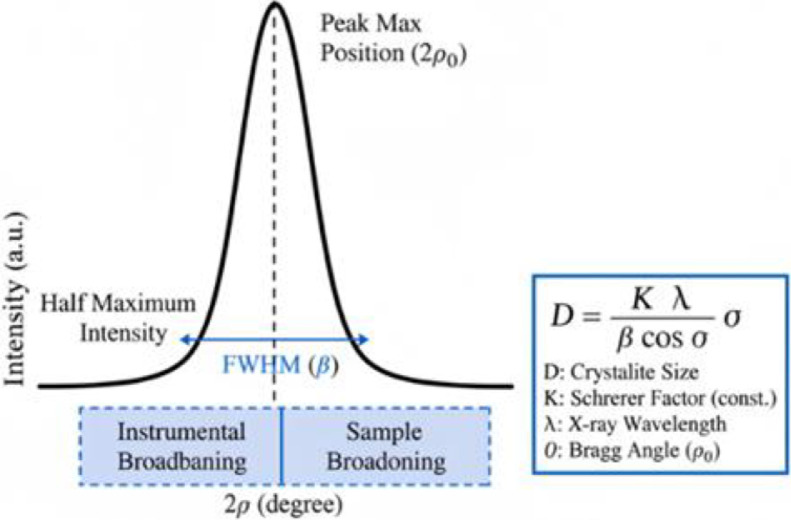
The width of an X-ray diffraction peak is related to crystallite size.

Calcined BPSA was kept in a desiccator (Secador® 1.0) to prevent moisture absorption. Phase analysis was carried out using XRD (Shimadzu XRD-7000) with reference to JCPDS card no. 26-1327. The Shimadzu XRD-7000 is an analytical instrument designed to study crystalline substances by identifying phases, examining crystal structures, and estimating crystallite size or internal stress by detecting diffracted X-rays at various diffraction angles (2θ) (Fig. 2).

The figure illustrates how the width of an X-ray diffraction peak is related to crystallite size through the Scherrer equation and how this width contains both instrumental and sample contributions. This diagram illustrates a typical X-ray diffraction (XRD) peak profile used to analyze crystallite size in powdered samples. It distinguishes between broadening effects from the instrument and the sample itself[12].

##### Peak profile basics

The curve plots intensity (in arbitrary units, a.u.) against the Bragg angle, 2θ (in degrees). A sharp peak reaches "Peak Max" at position 2θ, with "Half Maximum" lines marking the full width at half maximum (FWHM, denoted as β) [13].

##### Broadening types

The wider blue region labeled "Instrumental" represents baseline broadening from equipment factors like X-ray source profile, slit widths, and goniometer optics. The narrower orange "Sample" region overlay shows additional broadening due to finite crystallite size or lattice strain in the material.​ 

##### Scherrer equation

**Fig. 3:**
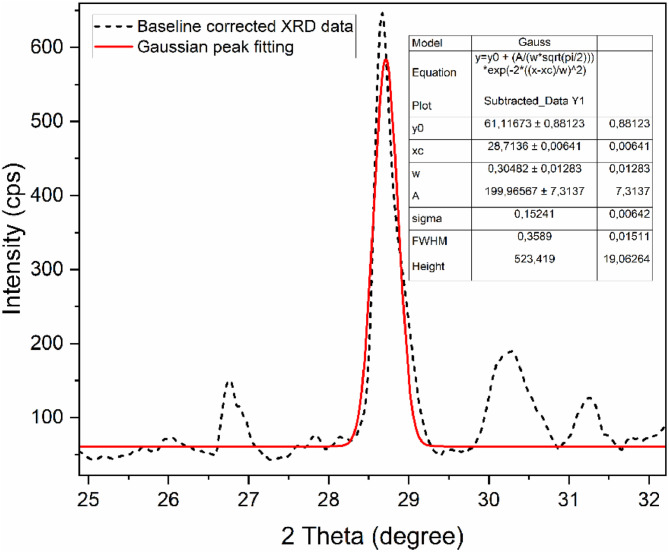
Peak fitting analysis of BPSA catalyst.

Fig. 3 presents the Gaussian peak fitting of the XRD spectrum for calcined BPSA catalyst at 2θ = 28.71°, performed using OriginPro-8 software. The diffractogram exhibits a high-intensity, narrow peak at this position, suggesting that the BPSA material possesses substantial crystallinity.

##### Extraction of peak parameters

From the fitted curve, the following parameters were obtained:​

• Peak position (2θ): 28.71°
• Full width at half maximum (FWHM): 0.38176°

These values serve as the essential inputs for crystallite size estimation via the Scherrer method. 

### Scherrer equation and assumptions

The Scherrer equation relates peak broadening to crystallite dimensions:​

(1) $$D=\frac{K\lambda }{\beta \text{cos}\theta }$$

where Dis the crystallite size, Kis the shape factor (typically 0.9), λis the X-ray wavelength, βis the FWHM in radians, and θis the Bragg angle. Standard assumptions include the use of Cu Kα radiation (λ = 0.15406 nm) and negligible instrumental broadening. 

### Calculation procedure

The Bragg angle is calculated as $\theta =28.71^{\circ }/2=14.355^{\circ }$, then converted to radians (0.2505 rad). The FWHM is similarly converted: $\beta =0.38176^{\circ }\;\times \;\left(\pi /180\right)=0.00666$rad. Substituting these values yields:​

(2) $$D=\frac{0.9\;\times \;0.15406}{0.00666\;\times \;\text{cos}\left(14.355^{\circ }\right)}=\frac{0.1387}{0.00645}\approx 22.9\;\text{nm}\;=\;23\;\text{nm}$$

### BET analysis

Prior to analysis, the calcined banana pseudostem ash (BPSA) samples were stored in a Secador® 1.0 desiccator to avoid moisture uptake. The catalysts were characterized by specific surface area, pore volume, and pore-size distribution using nitrogen adsorption–desorption analysis based on the Brunauer–Emmett–Teller (BET) method. Measurements were carried out using a surface area and porosity analyzer (e.g., Micromeritics ASAP 2020, USA). Approximately 0.2 g of each calcined sample was loaded into the analysis tube and degassed under vacuum at 200 °C for four hours to eliminate physically adsorbed moisture and volatile residues. After degassing, nitrogen gas was used as the adsorbate, and adsorption–desorption isotherms were recorded at liquid nitrogen temperature (77 K). The resulting isotherms depict the volume of nitrogen adsorbed at various relative pressures (P/P₀). The specific surface area of the catalyst was calculated from these data using the BET equation, which correlates the quantity of adsorbed gas to the accessible surface area of the solid. The BET equation is as follows:

(3) $$\frac{1}{v\left(\frac{P_{0}}{P}-1\right)}=\frac{C-1}{v_{m}C}\left(\frac{P}{P_{0}}\right)+\frac{1}{v_{m}C}$$

Where v is volume of gas adsorbed at pressure P, vₘ is monolayer adsorbed gas quantity, P/P₀ is relative pressure and C is BET constant related to the adsorption energy. The monolayer adsorption capacity vₘ) was determined from the linear portion of the BET plot within a relative pressure range of 0.05-0.30 and subsequently used to calculate the catalyst’s specific surface area (m² g⁻¹). The total pore volume was derived from the amount of nitrogen adsorbed at a relative pressure near saturation (P/P0≈0.99). Meanwhile, the Barrett-Joyner-Halenda (BJH) method, applied to the desorption branch of the isotherm, provided the pore size distribution and average pore diameter. These surface and textural parameters play a crucial role in determining catalytic performance, as they influence reactant diffusion and accessibility to active sites during transesterification.

#### Biodiesel production

**Fig. 4:**
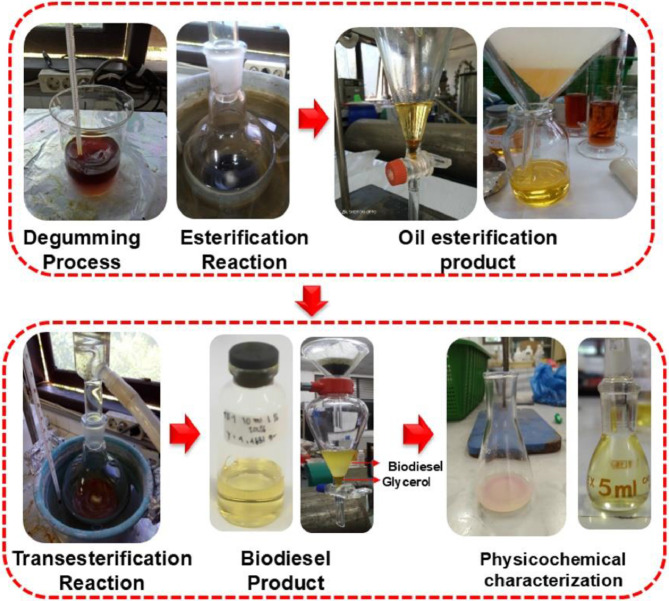
Image of the preparation stage of raw materials for the esterification-transesterification process.

Thirty grams of CPO were transesterified with methanol at molar ratios of 1:6, 1:8, 1:10, and 1:12 at 65 °C for reaction times of 60, 90, and 120 minutes. BPSA catalyst was added at loadings of 2, 4, and 6 wt%. After reaction, the mixture was separated using a separatory funnel and left to stand for 24 h, resulting in the formation of two layers: fatty acid methyl esters (FAME) and glycerol. Next, the yield obtained is calculated and the characteristics of the biodiesel are analyzed in accordance with biodiesel quality standards. The transesterification process is shown in Fig. 4.

The FAME layer was purified by dry washing in an oven at 80 °C for 12 h. The FAME yield was calculated and analyzed based on the Indonesian National Standard (SNI 7182:2015). The yield (wt%) was determined by dividing the mass of FAME (g) by the initial mass of CPO (g).

(4) $$Yield\;biodiesel\;\left(\%\right)\;=\;\frac{\text{weight}\;\text{of}\;\text{biodiesel}}{\text{weight}\;\text{of}\;\text{oil}}\;x100$$

### GC–MS analysis of FAME

The fatty acid methyl ester (FAME) profile of the biodiesel was determined using gas chromatography-mass spectrometry (GC–MS) (Shimadzu GCMS-QP2010, Kyoto, Japan) equipped with an Rxi-1ms capillary column (30 m × 0.25 mm i.d., 0.25 µm film thickness). Prior to injection, biodiesel samples were diluted with n-hexane at a 1:10 (v/v) ratio. A 1 µL aliquot of the diluted sample was introduced into the GC in split mode with a split ratio of 1:20, using helium as the carrier gas at a constant flow rate of 1.0 mL min⁻¹. The injector temperature was maintained at 250 °C. The oven temperature program was set as follows: initial temperature of 60 °C (held for 2 min), ramped to 200 °C at 10 °C min⁻¹, then increased to 280 °C at 5 °C min⁻¹ and held for 10 min. The mass spectrometer operated in electron ionization (EI) mode at 70 eV, with an ion source temperature of 200 °C and an interface temperature of 280 °C, and mass spectra were acquired over an m/z range of 40-500.

FAME components were identified by combining chromatographic and mass spectral information. Retention times of sample peaks were first compared with those of commercial FAME standards analyzed under identical GC conditions. Mass spectra of individual peaks were then matched against spectral libraries (e.g., NIST, Wiley) using the instrument software, and only hits with similarity indices above the acceptance threshold were considered positively identified. This dual approach-retention time comparison and library matching- provided reliable assignment of FAME structures. The relative content of each FAME was calculated from peak areas and expressed as a percentage of the total integrated FAME peak area in the biodiesel sample.

The resulting fatty acid profile governs key fuel properties such as kinematic viscosity, oxidative stability, and low-temperature flow behavior. A higher proportion of monounsaturated fatty acids (MUFA) is generally preferred because it improves oxidative stability while maintaining acceptable cold-flow characteristics, whereas excessive polyunsaturated tends to decrease oxidative stability and may deteriorate fuel quality. Feedstocks enriched in MUFA are therefore widely recommended for biodiesel production to achieve a favorable balance between storage stability and engine performance.

(5) $$Acid\;number\;=\;\frac{\left(A\;x\;N\right)}{W}x\;56,1$$

Where:

A = ml KOH required for titration of the sample
N = Normality of the KOH solution
W = grams of sample used
56.1 = molecular weight of KOH

#### Density measurements

Density was measured using a calibrated Pyrex pycnometer in accordance with ASTM D4052. Samples were carefully introduced, weighed with a digital balance (Shimadzu AW220), and density was obtained as the mass-to-calibrated-volume ratio at 15 °C. Density plays a crucial role in fuel injection, spray atomization, and energy content, so compliance with specification limits is required to ensure compatibility with modern diesel engines. Even small deviations in density can alter combustion behavior and emission characteristics, which makes precise determination essential for fuel quality evaluation.

(6) $$Biodiesel\;density=\;\frac{\text{mass}\;\text{of}\;\text{biodiesel}\;\left(g\right)}{\text{Volume}\;\text{of}\;\text{biodiesel}\;\left(\text{ml}\right)}$$

#### Kinematic viscosity

Kinematic viscosity was measured with a capillary viscometer at 40 °C following ASTM D445. This property determines how easily fuel flows through pumps and injectors and has a significant effect on atomization and spray pattern within the engine. Biodiesel with viscosity within the optimal range supports efficient combustion and reduces deposit formation. Changes in fatty acid chain length and degree of unsaturation directly influence viscosity-longer, saturated chains typically increase viscosity, while shorter, more unsaturated chains decrease it.

The kinematic viscosity of the biodiesel was calculated using the following equation:

(7) Kinematicviscosityofbiodiesel=C.t

Where: C = Capillary constant (cSt) t = Flow measurement time (seconds)

#### Cetane number

The cetane number was measured using a Cooperative Fuel Research (CFR) engine in accordance with ASTM D613. Cetane number characterizes the ignition quality of biodiesel in compression-ignition engines, where higher values correspond to shorter ignition delay and more complete combustion. The cetane response is closely linked to the fatty acid composition: saturated long-chain esters typically raise the cetane number, whereas high levels of unsaturation tend to reduce it. Meeting or exceeding the minimum cetane value specified in international standards is required to ensure reliable engine operation

Together, these analytical techniques form a comprehensive basis for evaluating the quality and fitness of biodiesel for application as a transportation fuel. By rigorously determining its fatty acid profile, density, viscosity, and cetane number, biodiesel can be verified not only for compliance with regulatory specifications but also for achieving an appropriate balance of performance, and environmental acceptability.

#### Experimental design for optimization of transesterification

The statistical design of experiments (DOE) is an effective approach for planning experiments that lead to reliable and objective conclusions based on data analysis. This study used experimental design to identify key factors affecting the process and determine their optimal conditions. Data regression and graphical analyses were performed using Design Expert (version 12.0.3.0) software (Stat-Ease Inc., Minneapolis, MN, USA). The Central Composite Design (CCD) a common response surface methodology (RSM), was applied for process optimization. Table 1 Design and analysis of experiments for the biodiesel production process using BPSA catalyst.

**Table 1:** Experiment design for biodiesel yield.

Run	Factor 1 Mol oil to methanol ratios (A)	Factor 2 Time (min)(B)	Factor 3 Mass catalyst (gr)(C)	Response 1 Actual yield (%) (Y)
1	1:8	120	4	95.8
2	1:5.6	107	5.2	97.0
3	1:8	60	4	96.5
4	1:8	90	4	98.0
5	1:4	90	4	98.1
6	1:8	90	2	97.4
7	1:8	90	4	98.1
8	1:8	90	6	97.2
9	1:10.4	107	5.2	97.6
10	1:10.4	72	5.2	98.4
11	1:5.6	72	2.8	97.9
12	1:10.4	72	2.8	97.3
13	1:12	90	4	97.6
14	1:5.6	107	2.8	96.8
15	1:8	90	4	98.0
16	1:8	90	4	98.0
17	1:5.6	72	5.2	97.2
18	1:8	90	4	98.0
19	1;8	90	4	98.0
20	1:10.4	107	2.8	97.6

The optimization aimed to find the best conditions for producing biodiesel from palm oil through the transesterification reaction. The independent variables were the molar ratio of oil to alcohol (A) and the transesterification time (B), and Mass Catalyst (C), While the response variable was the yield (Y), representing the percentage of biodiesel produced. The optimization analysis was conducted with Design Expert software version (version 12.0.3.0), which integrates statistical and mathematical techniques to design experiments, fit models, and determine the optimal operating conditions for a given response. In this study, the Central Composite Design (CCD) approach was applied, generating 20 experimental runs for one response variable, as shown in Table 1.

## Method validation

### Result interpretation of BPSA catalyst

As shown in Fig. 3, peak-fitting analysis of the BPSA catalyst yields an estimated crystallite size of about 23 nm. This nanoscale dimension is consistent with the sharp, well-defined diffraction peaks, confirming that calcination generated a material with a high degree of crystalline order. When reporting this parameter, it is appropriate to quote the crystallite size as approximately 23 nm to account for uncertainties in the fitting procedure and possible instrumental effects. 

**Fig. 5:**
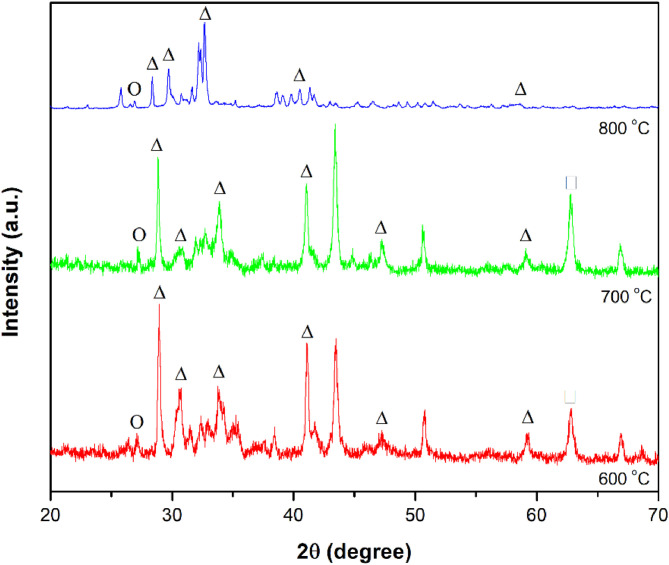
XRD pattern of BPSA at calcination temperature of 600°C and 700 °C.

Fig. 5 presents the X‑ray diffraction (XRD) patterns of banana pseudostem ash (BPSA) calcined at 600, 700, and 800 °C for 4 h. The samples display sharp, well‑defined reflections, confirming a highly crystalline structure with multiple mineral phases that provide catalytically active basic sites. The main diffraction peaks are assigned to potassium (△), calcium (◯), and magnesium (□) phases, based on JCPDS card 75‑078. Strong reflections at 2θ ≈ 28.3°, 33.7°, 40.5°, 45.6°, 58.7°, and 83.9° correspond to tetragonal potassium‑containing phases such as K₂O and K₂CO₃, while peaks near 27.3° and 56.5° are attributed to calcium‑ and magnesium‑based oxides. Several weaker, low-intensity peaks indicate the presence of phosphate, silica, carbonate, and minor Mg‑bearing phases, which further contribute to the material's overall basicity.

Raising the calcination temperature from 600 to 700 °C enhances crystallite growth and phase purity, as evidenced by the increased sharpness and resolution of the potassium‑dominated reflections. At 800 °C, however, the intensity of several peaks decreases and the patterns become slightly broader, suggesting partial sintering and coarsening of crystallites, which reduce the number of well‑defined diffracting domains and may lower the density of accessible active sites. This evolution indicates that, while moderate calcination improves crystallinity, excessively high temperatures can induce structural densification and compromise the optimal textural properties required for maximum catalytic performance [14].

**Fig. 6:**
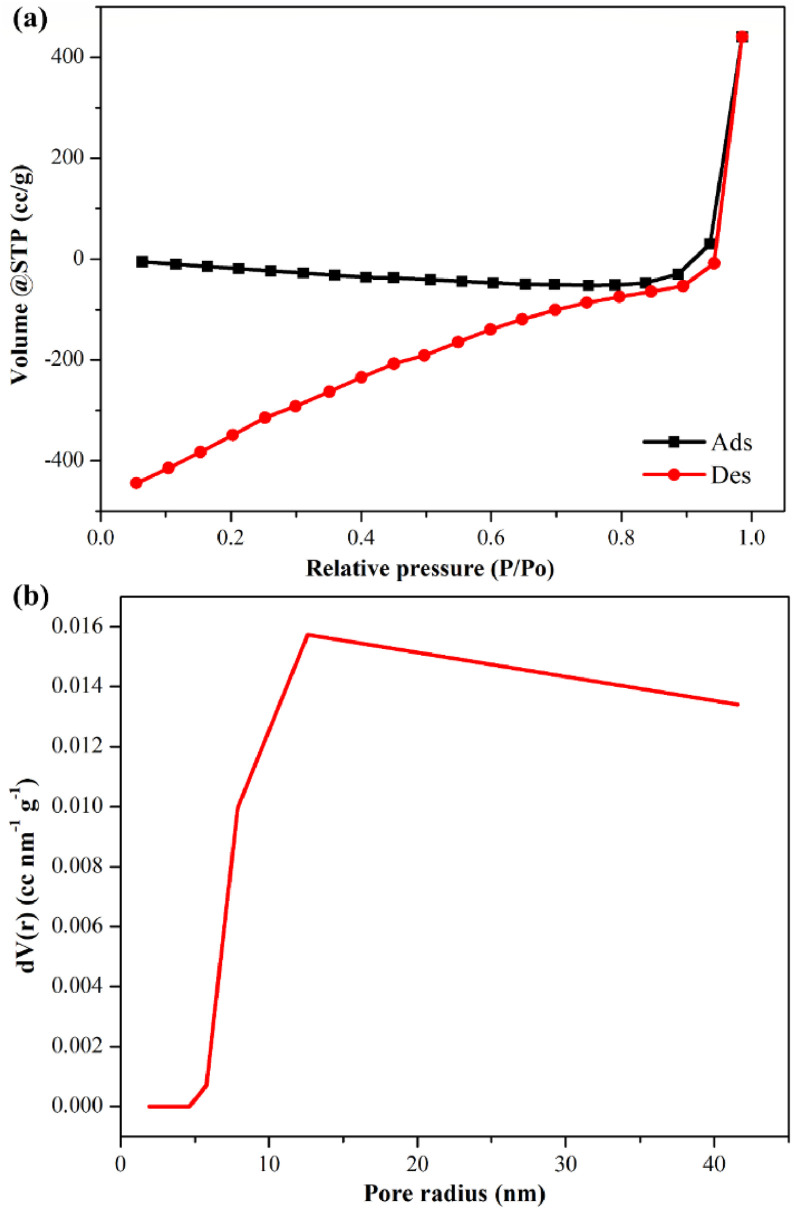
BET analysis result of BPSA(a) Adsorption-desorption isotherms (N2) and (b) catalyst pore size distributions.

Fig. 6 presents the BET analysis of the BPSA catalyst. The adsorption–desorption isotherm exhibits a typical type IV profile, indicating a mesoporous structure, consistent with previous reports by Laskar et al. [15] and Pathak et al [16]. The catalyst shows a specific surface area of 57.04 m²/g, a pore volume of 0.8196 cc/g, and an average pore radius of 12.61 nm, confirming its mesoporous nature.

The catalytic performance of heterogeneous base catalysts is strongly influenced by surface area and porosity [17,18]. These properties enhance mass transfer and provide accessible active sites during transesterification [19], leading to improved biodiesel yield and quality [20]. Therefore, the textural characteristics of the BPSA catalyst are considered sufficient to support effective catalytic activity, particularly due to the presence of alkaline active species.

### Biodiesel production: effect of oil-to-methanol ratio, catalyst loading, and reaction time

**Fig. 7:**
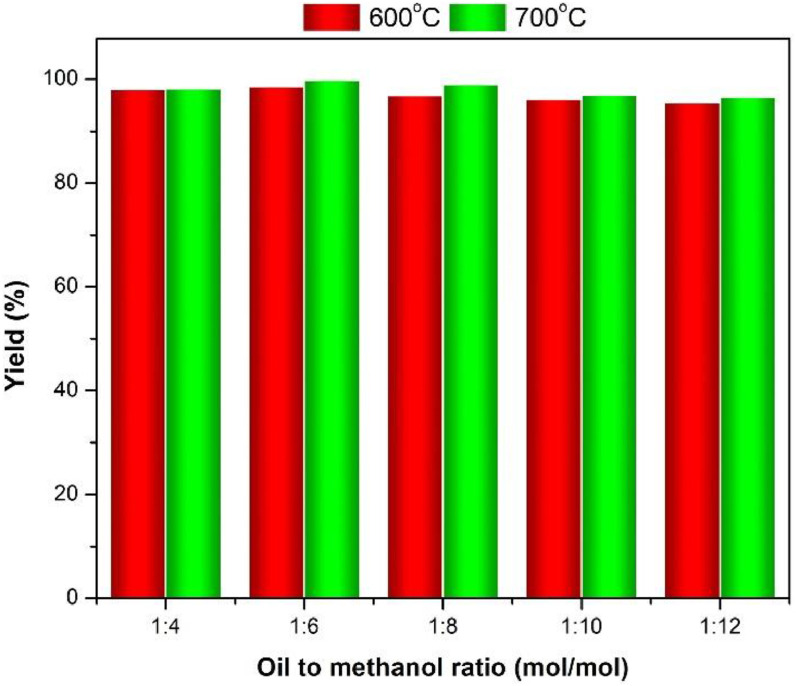
Impact of oil-to-methanol ratio on FAME production over BPSA catalysts calcined at 600°C and 700°C.

Transesterification of crude palm oil (CPO) to biodiesel using banana pseudostem ash (BPSA) catalysts calcined at 600 °C and 700 °C was carried out to identify optimal operating conditions, namely oil-to-methanol molar ratio, catalyst loading. Fig.7. shows that the CPO-to-methanol molar ratio is a decisive parameter governing biodiesel yield.

For both calcination temperatures, increasing the methanol amount initially enhances FAME production, with a maximum yield of 99.84 wt% obtained at a 1:6 CPO-to-methanol molar ratio. Further increases in methanol beyond this optimum reduce the yield, as excess methanol dilutes the reaction mixture, weakens contact between the catalyst and reactants, and increases glycerol solubility in the methanol phase, which complicates phase separation and lowers the effective conversion. These trends agree with previous observations for heterogeneous base-catalyzed systems (literature).

**Fig. 8:**
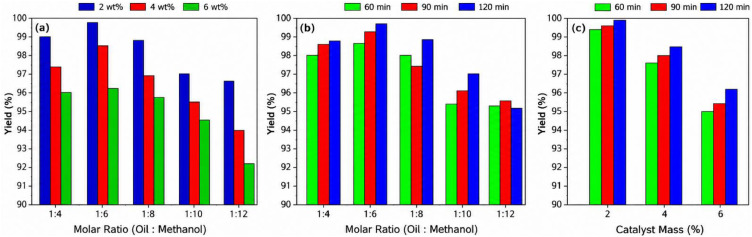
(a) Effect of molar ratio oil:methanol and BPSA catalyst loading; (b) Effect of molar ratio oil:methanol and reaction time (c) Effect of BPSA catalyst loading and reaction time at molar ratio oil:methanol 1:6 on FAME yield from crude palm oil.

The influence of catalyst loading (2wt%,4wt%, and 6 wt%) and reaction time (60 min, 90 min, 120 min) at a calcination temperature of 700 °C are illustrated in Fig. 8 (a-b). The highest FAME yield is consistently achieved at a catalyst dosage of 2 wt%, highlighting the need for an optimal catalyst amount. Reaction time also plays a crucial role, with 120 min giving the best conversion across the studied molar ratios, whereas extending the reaction beyond this point does not improve yield at higher methanol ratios because of the limitations.

Fig. 8(c) shows the effect of catalyst loading and reaction time on biodiesel yield at a methanol-to-oil molar ratio of 1:6 and a calcination temperature of 700 °C. At all reaction times (60, 90, and 120 min), the highest yield is obtained with 2 wt% catalyst, reaching to 99.84%, and then gradually decreases as the catalyst mass is increased to 4 and 6 wt%. This trend suggests that an optimum catalyst loading exists around 2 wt%, beyond which additional catalyst does not improve conversion and may introduce mass‑transfer limitations or increased mixture viscosity. Extending the reaction time from 60 to 120 min slightly increases the yield at each catalyst loading, indicating that a longer contact time favors completion of the transesterification. However, the effect of time is less pronounced than that of catalyst mass under these conditions.

Overall, the BPSA catalysts calcined at 600 °C and 700 °C enable very high biodiesel yields when the process variables are judiciously adjusted. Fig. 6 shows that a CPO-to-methanol molar ratio of 1:6 is optimal, while additional methanol beyond this point becomes detrimental to conversion. Fig. 8(a - c) indicates that a catalyst loading of 2 wt% combined with a reaction time of 120 min at this molar ratio gives the best performance. In contrast, higher catalyst dosages or unnecessarily long reaction times do not yield further gains and can slightly depress yield. These findings demonstrate that fine‑tuning the alcohol ratio, catalyst amount, and reaction time is critical for achieving near‑quantitative transformation of crude palm oil into biodiesel using BPSA catalysts.

### The physical and chemical properties of the biodiesel produced

The physicochemical quality of the produced biodiesel was evaluated based on the parameters measured in this study, namely density, FAME content, kinematic viscosity, cetane number, calorific value, and acid value, as summarized in Table 2.

**Table 2:** Biodiesel quality test parameters.

Parameters	Result	Indonesian Standard (SNI 7182: 2015)	ASTM D-6751 Biodiesel
Density (kg/m^3^) at 15^o^C	866	850 – 890	880 - 900
FAME (%wt, min)	99.84	96.5	-
Kinematic viscosity (mm^2^/s) at 40°C, (cSt)	3.26	2.3 – 6.0	1.9 – 6.0
Cetane number	63.6	Min 51	48 - 65
Calorific value (MJ/kg)	39.962	-	37 – 42.5
Acid Value (mgKOH/gr)	0.336	0.5	0.5

The produced biodiesel exhibited a pale-yellow color, clear appearance, and low viscosity, consistent with typical high-quality biodiesel. Its key physicochemical properties were as follows: density at 15 °C of 866 kg m⁻³, FAME purity of 99.84 wt%, kinematic viscosity at 40 °C of 3.26 cSt, cetane number of 63.6, calorific value of 39.96 MJ kg⁻¹, and acid value of 0.336 mg KOH g⁻¹. These values lie within the allowable ranges prescribed by SNI 7182:2015 and ASTM D6751 for biodiesel fuels. The data suggest that the transesterification proceeded efficiently and that the resulting fuel possesses favorable combustion-related characteristics. It should be noted, however, that comprehensive conformity with ASTM certification would require additional testing (e.g., flash point, cold-flow behavior, water content, and carbon residue), which was not included in this work. The free fatty acid (FFA) level, an important quality parameter for biodiesel obtained via base-catalyzed transesterification, can be inferred from the acid value. Using the measured acid value of 0.336 mg KOH g⁻¹, the FFA content was estimated to be approximately 0.169%, confirming a low residual FFA concentration in the final product. Furthermore, GC analysis confirmed the composition of fatty acid methyl esters (FAMEs), predominantly consisting of methyl hexadecanoate (58.37%), methyl oleate (30.94%), methyl linoleate (6.94%), methyl stearate (2.39%), and a minor fraction of methyl tetradecanoate (1.36%

Cetane number represents the tendency of a diesel fuel to auto-ignite under high pressure and temperature and is directly related to ignition delay, combustion noise, and exhaust emissions. Higher cetane numbers generally correspond to shorter ignition delays and more stable combustion. International biodiesel standards typically require minimum cetane numbers in the range of 47–51. The relatively high cetane number obtained in this work (63.6) suggests good ignition quality and is consistent with the fatty acid composition of the methyl esters present in the biodiesel.

## Results of operating condition optimization analysis

### Predictive model for CPO biodiesel yield

Model prediction and regression through ANOVA. Based on the results in Table 1, the program analysis (Design Expert), a number of experiments are recommended to be carried out (20 runs). The matrix design (experiment and prediction) of the analysis is shown in Table 3.

**Table 3:** Summary of the statistical model for biodiesel yield using BPSA catalyst.

Response	Sources	Standard deviation	R^2^	Adj-R^2^	R^2^ prediction
Biodiesel Yield	Linier	0.6920	0.0862	-0.0852	-0.5276
	2FI	0.7462	0.1368	-0.2616	-1.0676
	**Quadratic**	**0.4332**	**0.7762**	**0.5748**	**-0.8029**
	Cubic	0.3222	0.9257	0.7648	-15.3701

A statistical evaluation was conducted to identify the most suitable regression model for describing biodiesel yield as a function of the process variables. Several model structures were examined, namely linear, two-factor interaction (2FI), quadratic, and cubic models. The model selection procedure relied on key statistical metrics, including the coefficient of determination (R²), the adjusted R², the predicted R², and the standard deviation.

As summarized in Table 3, the quadratic model provides a better overall fit than the linear and 2FI models, with R² values of 0.7762 and 0.5748, respectively, indicating that it explains a moderate proportion of the variability in biodiesel yield. The superior performance of the quadratic model is mainly associated with the inclusion of second-order terms that account for curvature in the response surface.

Nevertheless, all evaluated models exhibit negative predicted R² values, reflecting limited predictive ability. This outcome suggests that the agreement between experimental and model-predicted yields is only moderate, possibly due to experimental noise, insufficient data, or the system's intrinsic complexity.

In light of these considerations, the quadratic model remains the most appropriate among the tested options for describing the influence of process variables on biodiesel yield and for interpreting factor interactions and overall trends, rather than for precise quantitative prediction.

**Fig. 9:**
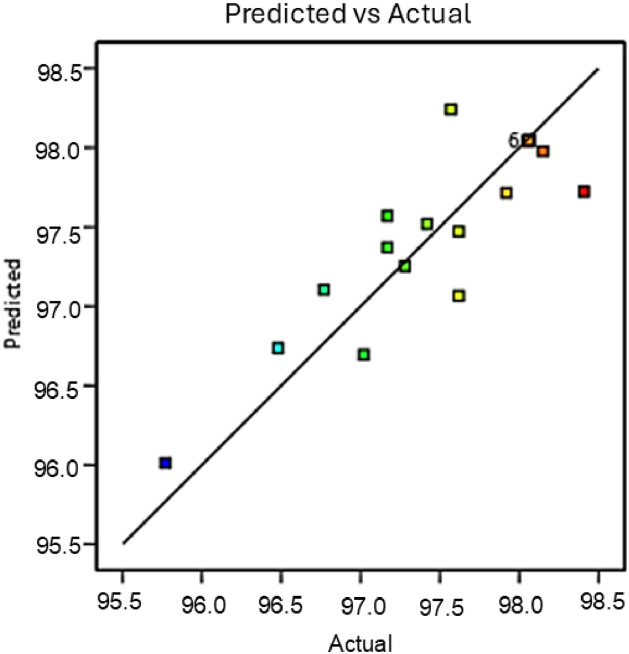
Relationship between actual data and predicted values of BPSA catalyst biodiesel yield.

Fig. 9 shows the relationship between the predicted and actual biodiesel yields using the BPSA catalyst, providing a visual view of the regression model’s performance. At lower actual yields (around 97–98.5%), the data points lie above the diagonal line, indicating that the model tends to overestimate these values. In contrast, at higher yields (above 99%), the points align more closely with the diagonal line, showing better prediction accuracy. This suggests that the model’s response is inconsistent across the data range, with systematic bias at certain yield levels. The misalignment also indicates unequal residual variance, which may affect the model’s reliability [21]

A statistical assessment confirmed that the quadratic model was the most suitable among the tested alternatives, with R² = 0.7762 and adjusted R² = 0.5748, indicating a moderate capacity to describe the variability in biodiesel yield. However, the negative predicted R² value (–0.8029) indicates poor predictive performance and limited model generalizability. This weakness is also apparent in Fig. 9, where substantial deviations from the parity line indicate weak agreement between the predicted and experimental responses. Moreover, the systematic pattern of residuals, marked by a consistent overestimation across the data range, suggests the presence of intrinsic model bias rather than purely random experimental error.

In general, interactions between variables can be described using mathematical equation models, such as the quadratic model used in this study. This equation can also be used to predict yield values (Table 4). The mathematical model is shown in Eq. (8).

(8) $$Y\;=\;85.85499\;-\;0.544003A\;+\;0.305235\;B\;+\;0.508455\;C\;+\;0.072037\;\text{AC}\;+\;0.002504\;\text{AB}\;-\;0.000766\;\text{BC}\;+0.003972A^{2}\;-\;0.001857\;B^{2}\;-\;0.125363\;C^{2}$$

**Table 4:** Validation of model prediction results on experimental data on biodiesel yield using BPSA catalyst.

Run	Mol methanol to oil ratios	Time (min)	Mass Catalyst	Yield Biodiesel		
			(gr)	Prediction	Experiments	Error
1	1:8	90	4	98.05	98.06	0.0143
2	1:5.6	72.2	2.8	97.72	97.92	0.2047
3	1:10.4	72.2	5.2	97.72	98.41	0.6867
4	1:5.6	107.8	5.2	96.69	97.02	0.3252
5	1:5.6	72.2	5.2	97.37	97.17	-0.2010
6	1:8	90	4	98.05	98.06	0.0143
7	1:8	90	4	98.05	98.06	0.0143
8	1:5.6	107.8	2.8	97.10	96.77	-0.3341
9	1:10.4	107.8	2.8	97.07	97.62	0.5535
10	1:8	90	4	98.05	98.06	0.0143
11	1:8	90	2	97.52	97.42	-0.0985
12	1:4	90	4	97.98	98.15	0.1730
13	1:8	60	4	96.74	96.48	-0.2568
14	1:8	120	4	96.01	95.77	-0.2417
15	1:12	90	4	98.24	97.57	0.6716
16	1:8	90	4	98.05	98.06	0.0143
17	1:10.4	72.2	2.8	97.25	97.28	0.0274
18	1:8	90	6	97.57	97.17	-0.4001
19	1:8	90	4	98.05	98.06	0.0143
20	1:10.4	107.8	5.2	97.47	97.62	0.1478

Statistically, this trend is consistent with the results of the previous quadratic model evaluation, which showed an R² value of 0.8195 and an Adjusted R² of 0.6905, indicating that the model is quite good at explaining data variation. However, the negative predictive R² value (–0.2836) makes it clear that the model fails to provide reliable estimates for new data. This situation often arises as a result of overfitting, where the model adapts too closely to the training data, thereby losing its ability to generalize. In addition, the color distribution in the graph, which represents different treatments, shows that prediction bias occurs across treatments, not just limited to one or two conditions.

Where,

Y = biodiesel yield
A = mol oil to methanol ratio
B = time
C = catalyst
AB = interaction of the mol ratio of methanol oil with time
AC = interaction of the mol ratio of methanol oil with catalyst
BC = interaction of the time with catalyst

Referring to Eq. (8), the effect of each parameter involved in the transesterification process can be identified. The positive and negative coefficients in the equation represent the synergistic and antagonistic relationships among the variables. Furthermore, by applying the developed model equation, the interaction between input factors and response variables can be visualized using a linear versus probability plot and a plot comparing experimental data with predicted values, as illustrated in Fig. 9.

The ANOVA results in Table 5 show that the overall regression model is statistically significant (p = 0.0235), indicating that the selected factors jointly affect biodiesel yield. However, a more detailed inspection of the individual terms reveals that this significance is not evenly shared across all variables. The linear effects of molar ratio (A), catalyst mass (B), and reaction time (C) are not statistically significant (p > 0.05) and exhibit low F-values, suggesting a limited direct impact within the investigated range. Even reaction time (C), which has a comparatively higher F-value (3.35), remains insignificant (p = 0.0958), indicating only a marginal effect.

**Table 5:** ANOVA biodiesel yield with BPSA catalyst.

Sources	Sum of square	DF	*Mean Square*	F-*Value*	*P-Value*	Keterangan
Model	6.51	9	0.7231	3.85	0.0235	*Significant*
A-Mol ratio	0.0845	1	0.0845	0.4505	0.5173	
B-Time	0.0032	1	-.0032	0.0171	0.8985	
C-Catalyst mass	0.6347	1	0.6347	3.38	0.0958	
AB	0.3321	1	0.3321	1.77	0.2130	
AC	0.0903	1	0.0903	0.4813	0.5036	
BC	0.0021	1	0.0021	0.0113	0.9176	
A^2^	0.0073	1	0.0073	0.0388	0.8479	
B^2^	0.4530	1	0.4530	2.41	0.1513	
C^2^	5.03	1	5.03	26.82	0.0004	
*Residual*	1.88	10	0.1877			
*Lack of Fit*	1.88	5	0.3753			
*Pure error*	0.0000	5	0.000			
*Cor Total*	8.38	19				

By contrast, the quadratic term of reaction time (C²) is highly significant (p = 0.0004; F = 26.82), demonstrating that the system is strongly governed by nonlinear behavior associated with this variable. This implies that biodiesel yield is predominantly influenced by curvature effects rather than by linear or interaction terms. All interaction terms (AB, AC, BC) and the remaining quadratic terms (A² and B²) are statistically insignificant (p > 0.05), indicating that multivariable interactions and secondary nonlinearities contribute little to the response. The relatively large residual contribution points to unexplained variability, likely arising from experimental noise or unmodeled factors, while the absence of pure error further constrains rigorous model validation. Collectively, these observations suggest that, although the model is globally significant, its explanatory power is heavily reliant on a single dominant quadratic term, consistent with its limited predictive robustness.

### Effect of the oil-to-methanol molar ratio and reaction time on biodiesel yield

**Fig. 10:**
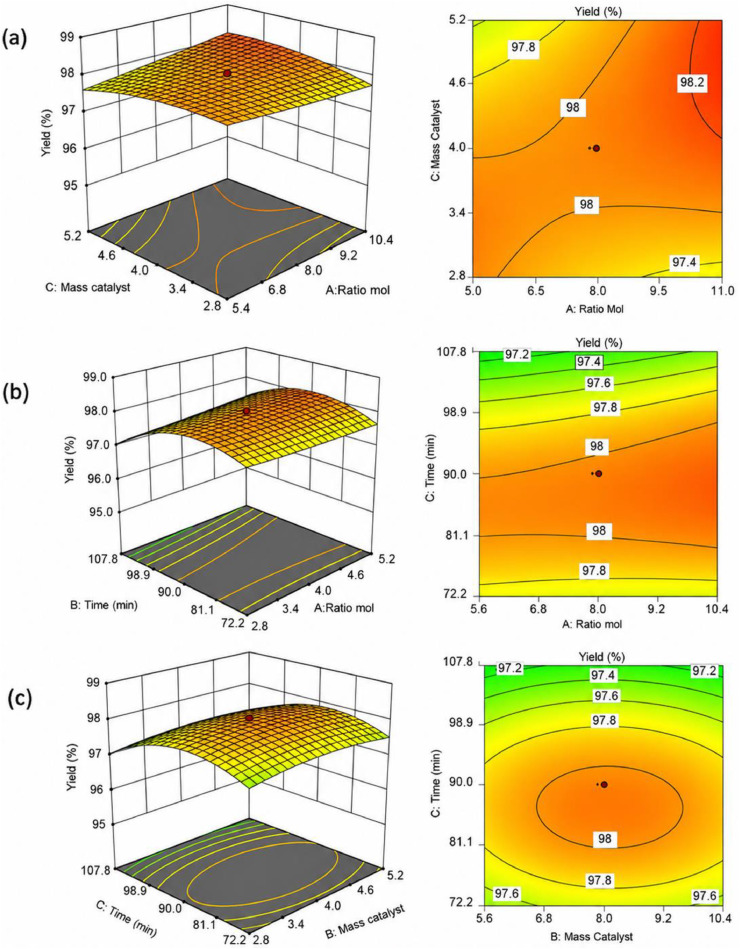
Contour and 3D graph interaction of methanol-oil molar (a) methanol-oil molar and and catalyst weight (b) methanol-oil molar and and reaction time (c), and catalyst weight and reaction time.

In the transesterification process, the key factors influencing the reaction are the oil-to-methanol molar ratio and the reaction time, both of which have a significant impact on biodiesel production. To examine the interaction between these variables and their effect on the resulting yield, the Response Surface Methodology (RSM) was applied. The 3D response surface and contour plots generated from this analysis illustrate the interaction between the oil-to-methanol molar ratio and reaction time on biodiesel yield. These plots are presented in Fig. 10.

The three-dimensional response surfaces and contour plots in Fig. 10(a-c) depict the combined effects of oil-to-methanol molar ratio (A), catalyst loading (B), and reaction time (C) on biodiesel yield using the banana corm ash catalyst. The 3D plots show that biodiesel yield initially increases with increasing molar ratio up to an optimum, beyond which the yield gradually declines, confirming a nonlinear relationship in line with the ANOVA results on curvature effects. Fig. 10(a) illustrates the interaction between catalyst loading and methanol-to-oil molar ratio, where a maximum yield of 98.4% is obtained at a molar ratio of 1:10.4. Further increasing catalyst loading above 5.2% at this molar ratio causes a slight reduction in yield, indicating that excess catalyst does not enhance, and may even hinder, the process.

Fig. 10(b) shows that an optimal reaction time of 105 minutes yields 98.4% biodiesel; extending the reaction to 120 minutes at constant catalyst loading results in a modest decrease in yield, suggesting that unnecessarily long reaction times reduce process efficiency. As shown in Fig. 10 (c), the highest yield (98.4%) is achieved under the combined optimal conditions of 10.4:1 methanol-to-oil molar ratio and 5.2% catalyst loading. Beyond this region, extending the reaction time at a fixed catalyst dosage results in lower yields, confirming the existence of a narrow optimal operating window for the system [22,23].

### The effect of the methanol oil ratio on palm oil biodiesel yields

**Fig. 11:**
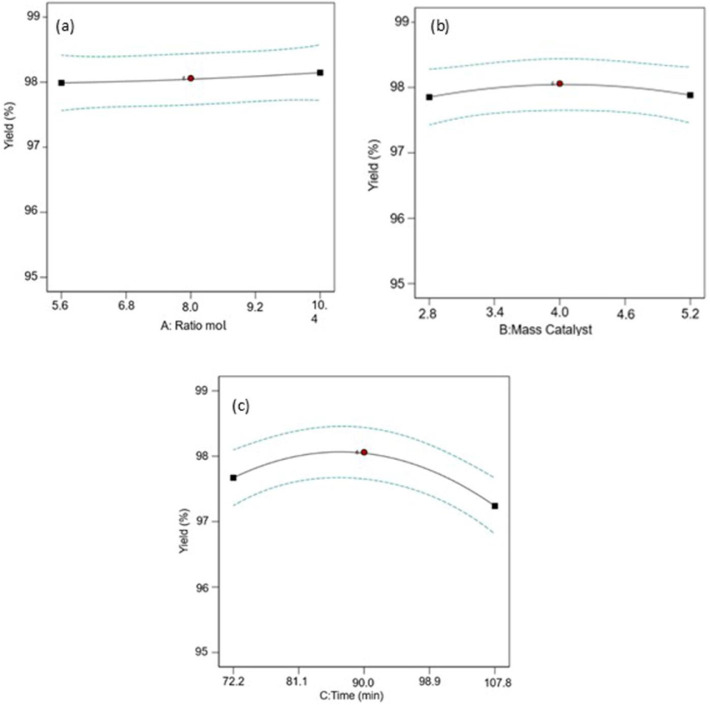
Effect of process parameters (a) methanol to oil molar ratio, (b) catalyst weight, and (c) reaction time.

The alcohol-to-oil molar ratio is a critical parameter in biodiesel production because, in a reversible transesterification reaction, an excess of alcohol drives the equilibrium toward ester formation. As shown in Fig. 11(a), increasing the molar ratio from about 6:1 to 10:1 resulted in only a slight increase in biodiesel yield, from roughly 98.0% to 98.2%, indicating that this variable has only a minor effect within the investigated range.

At higher alcohol loadings, excess ethanol can increase glycerol solubility and hinder phase separation, thereby lowering the effective biodiesel yield. Conversely, an insufficient alcohol ratio may limit conversion because of mass-transfer constraints between the oil and alcohol phases. In the present work, changes in the methanol-to-oil molar ratio produced only marginal improvements in yield, and the factor was statistically insignificant (p > 0.05), suggesting that, over the studied interval, the process is relatively insensitive to this parameter and that other variables exert a stronger influence on biodiesel production

### The effect catalyst weight on palm oil biodiesel yields

The effect of catalyst loading on biodiesel yield, as presented in Fig. 11(b), exhibits a distinctly nonlinear trend. The yield increases slightly from about 97.8% to 98.0% when the catalyst dosage is raised from 3% to 4%, but then decreases when the loading is further increased to around 5%, indicating an optimum catalyst concentration beyond which additional catalyst does not enhance conversion. This behavior is commonly attributed to increased mixture viscosity and mass‑transfer resistance, as well as the possible promotion of side reactions, such as saponification, at higher catalyst contents, all of which can lower the effective biodiesel yield.

The initial yield enhancement can be attributed to the greater number of available active sites, whereas excessive catalyst loading tends to increase mixture viscosity and introduce mass-transfer limitations [24]. In addition, excess catalyst may favor side reactions, such as saponification, thereby lowering the effective biodiesel recovery [25,26]. Comparable trends have been reported in previous studies, in which overdosing the catalyst ultimately reduces conversion efficiency [27]

### The effect reaction time on palm oil biodiesel yields

The effect of reaction time on biodiesel yield, as presented in Fig. 11(c), also follows a nonlinear trend. The yield increases from about 97.7% at 75 min to a maximum of roughly 98.0% at 90 min, showing that providing sufficient reaction time promotes the progress of the transesterification. When the reaction is prolonged to around 105 min, however, the yield decreases noticeably, indicating that overly long reaction times do not further improve the overall efficiency of the process and may even impair it.

The initial rise in yield can be linked to improved interaction between reactants and the system’s approach to equilibrium. In contrast, prolonged reaction time may intensify reversible reactions or side reactions, such as hydrolysis and saponification, thereby diminishing the amount of ester formed. Extended exposure can also promote partial product degradation or increased glycerol solubility in the ester phase, thereby lowering the effective biodiesel recovery [25]. Consistent with previous reports, biodiesel yield typically increases up to an optimal reaction time and then declines due to equilibrium constraints and secondary reactions [28,29]. Thus, reaction time is a critical operating variable with a distinct optimum, beyond which overall process performance deteriorates.

## Determination of optimum conditions

### Optimum conditions for banana pseudostem ash (BPSA) catalyst

The simultaneous optimization of response variables can be performed using the Desirability Function (DF) approach. The Design-Expert software version 12.0.3.0 provides a built-in feature for conducting process optimization through the DF method. The parameter values for each independent variable as well as the response variable are presented in Table 6.

**Table 6:** Parameter values of the independent variables and the response variable used for process optimization.

Component	Goal	Lower	Upper
Mole ratios	in range	4.58579	7.41421
Time	in range	68.7868	111.213
Yield	maximize	97.8	99.845

Table 6 presents the optimized process parameters for the transesterification reaction in biodiesel production using a BPSA catalyst. The optimization was carried out for two independent variables, namely the methanol-to-oil molar ratio and the reaction time, with the objective of maximizing the response variable, i.e., methyl ester yield (%). The optimization model was developed using the statistical framework of Response Surface Methodology (RSM), which is widely applied in process engineering to efficiently determine the best operating conditions. The optimization calculations focused on achieving the maximum possible yield (Y), as targeted in the design criteria. Using Design-Expert version 12.0.3.0, the optimization procedure produced a single optimal solution, which is reported in Table 7.

**Table 7:** Several alternative optimum conditions recommended by Design-Expert.

No	Mol ratio	Time	Yield	Desirability	
1	6.249	104.558	99.558	0.888	Selected
2	6.248	104.375	99.558	0.888	

Table 7 presents two alternative optimum conditions for the methanol-to-oil molar ratio and reaction time that yield high biodiesel production, along with the corresponding desirability values as a combined indicator of the optimization criteria. To select the most suitable alternative, the highest response variable (yield) and the desirability function (DF) value closest to 1 are considered. The desirability value reflects how well a given solution meets the optimization goals; on a 0–1 scale, a value of 0.888 indicates that the selected condition is very close to ideal for achieving the specified target, namely maximizing biodiesel yield while satisfying the constraints on the other variables.

**Fig. 12:**
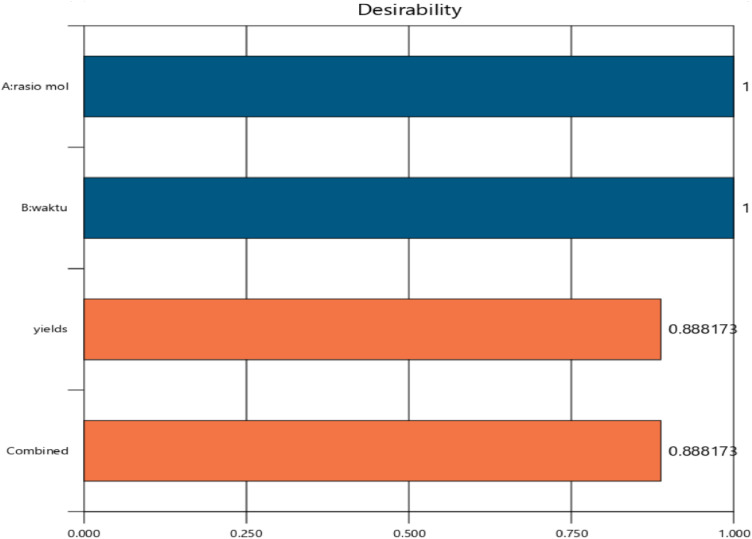
Desirability of the methanol-to-oil molar ratio and reaction time on biodiesel yield.

The desirability profiles for the oil-to-methanol molar ratio and reaction time are shown in Fig. 12.

As the final stage of the optimization process, a desirability analysis was carried out to assess how well the obtained parameters satisfy the predefined objective of maximizing biodiesel yield within the allowable ranges of the oil-to-methanol molar ratio and reaction time. The results of this analysis are illustrated in Fig. 12, which displays the desirability values for each input variable (molar ratio and reaction time) as well as for the output variable (biodiesel yield).

Fig. 12 shows that both the molar ratio and reaction time variables have desirability values of 1.000. This indicates that the optimized parameters, namely a molar ratio of 6.249 and a reaction time of 104.558 minutes, lie exactly within the predefined acceptable ranges. This result confirms that both variables satisfy the in-range criterion specified in the optimization strategy, without requiring either a maximum or minimum extreme value.

Unlike the process parameters, biodiesel yield is defined as the response variable that must be maximized. Under the obtained optimum conditions, the biodiesel yield reaches 99.56%, which is very close to the theoretical maximum within the optimization range, namely 99.85%. Although it does not attain the absolute maximum, this yield is still very high and corresponds to a desirability value of 0.888. This indicates that the system has converted oil to methyl esters with very high efficiency, with only minimal deviation from the ideal target.

The combined desirability value, obtained from the aggregation of the individual desirability functions for all variables, is also 0.888. In the context of response modeling and process optimization, a combined desirability above 0.8 is generally regarded as very good and reflects strong agreement between the input variables and the response with respect to the optimization goals. This further supports the selection of these process conditions as the optimum, not only statistically valid but also practical for implementation on a larger scale. Overall, this evaluation strongly confirms that the identified optimum conditions can deliver excellent process performance, characterized by high yield and stable parameters within realistic control limits. In addition, the consistency of desirability values across the alternative suggested conditions indicates that the system is robust to small fluctuations in process parameters, which is crucial for large-scale and sustainable industrial applications [30]

The combined desirability value, obtained from the aggregation of the individual desirability functions for all variables, is also 0.888. In the context of response modeling and process optimization, a combined desirability above 0.8 is generally regarded as very good and reflects strong agreement between the input variables and the response with respect to the optimization goals. This further supports the selection of these process conditions as the optimum, not only statistically valid but also practical for implementation on a larger scale. Overall, this evaluation strongly confirms that the identified optimum conditions can deliver excellent process performance, characterized by high yield and stable parameters within realistic control limits. In addition, the consistency of desirability values across the alternative suggested conditions indicates that the system is robust to small fluctuations in process parameters, which is crucial for large-scale and sustainable industrial applications [31].

## Catalyst reusability

**Fig. 13:**
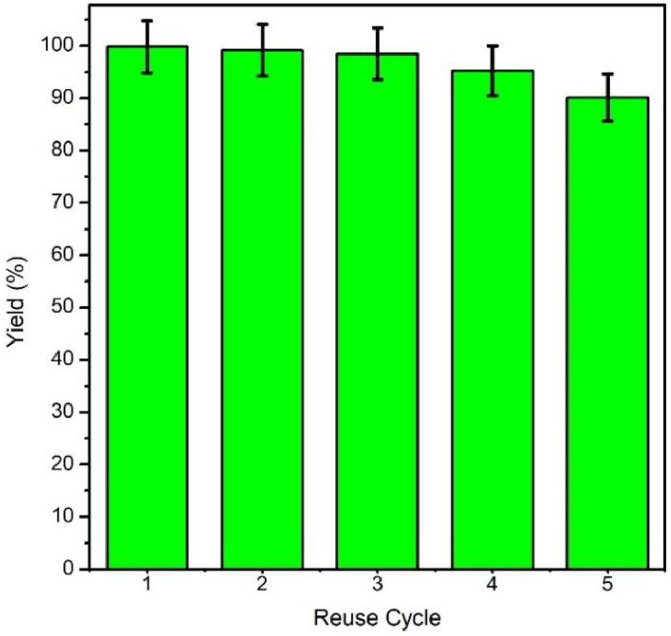
Effect of reuse cycles on biodiesel yield over the BPSA-derived K₂O catalyst.

Under the optimized reaction conditions (methanol to oil molar ratio 1:6, calcination temperature 700 °C, reaction time 120 min, and catalyst loading 2 wt%), the reusability of the BPSA catalyst is summarized in Fig. 13. The biodiesel yield decreases from 99.84% in the first cycle to 99.14% and 98.50% in the second and third cycles, followed by more pronounced drops to 95.23% and 90.11% in the fourth and fifth cycles, respectively. The error bars in Fig. 13 represent the standard deviation of repeated measurements and indicate that the experimental results are reasonably reproducible across all reuse cycles. Relatively small error bars in the first three cycles suggest stable catalytic performance with low experimental variation, whereas the slightly wider variation observed in the later cycles may reflect increasing instability of the catalyst as deactivation progresses. Overall, the decline of about 9.7 percentage points across five runs indicates progressive catalyst deactivation, consistent with typical behavior reported for solid base catalysts in transesterification.

The loss of activity can mainly be ascribed to three deactivation mechanisms. First, leaching of soluble potassium-containing species from the BPSA surface into the reaction medium reduces the number of strong basic sites available in subsequent cycles, as commonly observed for K₂O/CaO based and other alkaline earth catalysts [30,31]. Second, surface fouling and pore blocking by adsorbed triglycerides, glycerol, soaps, and other by products progressively hinder reactant access and lower the effective surface area. Third, repeated reaction, washing, and drying steps may induce partial sintering or structural rearrangement of the ash particles, decreasing dispersion and basic site density. Similar trends in high initial FAME yield followed by a marked decline with reuse due to leaching, fouling, and structural changes have been widely documented for biomass-derived heterogeneous biodiesel catalysts[14,32].

## Related research article

None.

## For a published article

None.

## Ethics statements

The work does not involve studies with animals and humans.

## CRediT author statement

Conceptualization, Methodology, Investigation, Writing original draft by Meriatna. Conceptualization, Validation, Data curation, Writing-review and editing, Supervision by Husni Husin. Validation, Data curation by Medyan Riza, Writing-review and editing, Supervision by M. Faisal and Pocut Nurul Alam, Investigation, Formal Analysis, Writing-review and editing by Fikri Hasfita. Validation, Data curation by Yuliana Sy and Leni Maulinda. Writing-review and editing by Muhammad Lathiful Yazil.

## Declaration of competing interest

The authors declare that they have no known competing financial interests or personal relationships that could have appeared to influence the work reported in this paper.

## Data Availability

Data will be made available on request.
